# Biogeographical role of the Kuroshio Current in the amphibious mudskipper *Periophthalmus modestus* indicated by mitochondrial DNA data

**DOI:** 10.1038/srep15645

**Published:** 2015-10-28

**Authors:** Lijun He, Takahiko Mukai, Ka Hou Chu, Qiang Ma, Jing Zhang

**Affiliations:** 1 State Key Laboratory of Estuarine and Coastal Research, East China Normal University, 200062, Shanghai, China; 2 East China Sea Fisheries Research Institute, Chinese Academy of Fishery Sciences, 200090, Shanghai, China; 3 Faculty of Regional Studies, Gifu University, 1-1 Yanagido, 501-1193, Gifu, Japan; 4 Simon F. S. Li Marine Science Laboratory, School of Life Sciences, The Chinese University of Hong Kong, Hong Kong, China; 5 Shanghai Chongming Dongtan National Nature Reserve, 202183, Shanghai, China

## Abstract

Quaternary climatic cycles have influenced marine organisms’ spatial distribution and population dynamics. This study aimed to elucidate the evolutionary influences of contemporary and glacial physical barriers on the population structure, demography and colonization history of the mudskipper (*Periophthalmus modestus*) based on a mitochondrial gene segment (ND5) from 131 individual fish sampled in the northwestern Pacific Ocean. The current Kuroshio Current and the glacial exposure of the Taiwan Strait appeared to have restricted migration among the South China Sea, coastal East China and Japan. However, genetic homogeneity (Nm>1) also suggested contemporary larval transportation by sea circulation between the East China Sea and the South China Sea or historical dispersal along the glacial exposed shoreline among China, Japan and the Ryukyu Islands. Evolutionary signals of the strengthened East Asian Summer Monsoon in the mid-Pleistocene and regional difference in intertidal primary productions were indicated by a late-Pleistocene population expansion of *P. modestus* with a higher effective population size in the South China Sea than in the East China Sea. Furthermore, a potential colonization origin from the South China Sea was consistently inferred by different clues, including the populations’ coalescence times, the ancestral haplotype distribution, the number of private haplotypes and species/genetic diversity.

Historical climatic changes are believed to have greatly influenced the coastal environment^1^ and the evolutionary history of its biota^2^,^3^. With periodic sea-level fluctuations, marine organisms have experienced repeated habitat expansions and contractions. These historic events were imprinted in the evolutionary processes, affecting the distribution and dynamics of populations^4^,^5^. It has been hypothesized that a rising sea level and range expansion could result in genetic homogeneity and rapid population growth^6^,^7^,^8^,^9^, whereas a lowering sea level and habitat fragmentation could lead to a heterogeneous population structure^4^ and a genetic bottleneck for many marine organisms^10^.

The marginal seas of the northwestern Pacific Ocean (such as Indonesian seas, China seas, and the Sea of Japan/East Sea) have attracted considerable attention in phylogeographical studies due to their complicated topography^1^,^11^ and high genetic diversity^12^,^13^. China seas (including the South China Sea, the East China Sea, the Yellow Sea and Bohai) provide a good dynamic physical model with which to test the evolutionary responses of marine organisms to the periodic geomorphologic and oceanographic changes during the Quaternary (Figs. 1, 2). The isolated glacial sea basins (viz. semi-enclosed South China Sea and the narrow Okinawa Trough), due to the exposure of the shallow continental shelves^14^ (Fig. 1a) and sub-habitats divided by the branching Kuroshio Currents (Fig. 1b), likely separated marine species into different populations, so lineage diversification and population differentiation could be expected (Fig. 2a,b). Nevertheless, these isolated populations would be remixed due to absence of the Kuroshio Current in the glacial semi-closed Okinawa Trough (Fig. 1a) or by postglacial coastal sea circulations driven by East Asian Monsoons between the East China Sea and South China Sea (Fig. 1b). Thus, a genetic homogeneous population could also be expected for some marine species in the China seas (Fig. 2c,d).

Several previous studies on different species showed the two above-mentioned contrasting evolutionary patterns in the China seas^15^. Deep lineage differentiation or heterogeneous populations (Fig. 2a,b) were observed in turban shell (*Turbo cornutus*)^16^, *Pandaka* gobies^17^, tideland snail (*Cerithidea cingulata*)^18^, mudskipper (*Boleophthalmus pectinirostris*)^19^, mullet (*Chelon haematocheilus*)^20^, acorn barnacles (*Tetraclita squamosa* and *T. japonica*)^21^,^22^, and mitten crabs (*Eriocheir sensu stricto*)^23^,^24^. Yet another pattern of population expansion with genetic homogeneity (Fig. 2c,d) was also found in tideland snail (*Batillaria zonalis*)^25^, spotted sea bass (*Lateolabrax maculatus*)^26^, Japanese anchovy (*Engraulis japonicus*)^27^, demersal fish *Nibea albiflora*^28^, neon damselfish (*Pomacentrus coelestis*)^29^, swimming crab *Portunus trituberculatus*^30^ and mud crab *Scylla paramamosain*^31^. The two contrasting phylogeographic patterns among these species could be attributed to their different evolutionary histories or dispersal capabilities. Therefore, to reveal the general evolutionary consequence of eustatic oscillations in the China seas, studies on phylogeographic histories of different species are highly desirable.

As other gobies are^13^,^17^,^32^,^33^, mudskipper *Periophthalmus modestus* (Cantor, 1842; Gobioidei: Gobiidae) is a good species for studying the evolutionary effects of sea-level fluctuations and physical barriers in the coastal northwestern Pacific Ocean because of its amphibious life history, which depends on the intertidal mudflat habitat^34^,^35^ and limited adult dispersal capability. This species is endemic in this region, including the coasts of the China seas, the Korean peninsula and Japan^36^. Mukai and Sugimoto^37^ found two divergent lineages and a significant genetic differentiation of *P. modestus* between the main islands of Japan (Honshu and Kyushu) and the Ryukyu Islands (Tanegashima and Okinawajima), but they could not elucidate the overall phylogeographic history of this species because of lack of samples from the Asian continental coast. In the present study, sequences of a mitochondrial gene from *P. modestus* along the coast of China were combined with those from the previous study to address three evolutionary questions: first, the evolutionary influence of barriers (e.g., the glacial exposure of the Taiwan Strait and contemporary branches of the Kuroshio Current) on population genetic structure; second, the demographic response to glacial cycles; and third, the colonization origin for this species in the China seas. This study will shed light on the understanding of relationships among Pleistocene climatic cycles, oceanographic conditions and the evolution of marine organisms.

## Results

### Phylogenetic analyses

A dataset with 76 haplotypes (772 bp) was obtained through sequence alignment. No indels or stop codons were found. In contrast to Mukai’s and Sugimoto’s result^37^, no different lineages or subspecies were identified in *P. modestus* through phylogenetic analyses (Fig. 3). A single lineage and a consistent branching pattern with a South China Sea haplotype (N66) basal to all other haplotypes was revealed in the neighbour-joining (NJ), maximum likelihood (ML) and Bayesian inference (BI) trees, although nodal supports for grouping the haplotypes other than N66 were not strong (BP = 62/58 for NJ/ML and posterior probability (PP) = 0.54 for BI). Likelihood mapping analysis corroborated the internal branch of the ingroup, excluding N66 with a strong support value (99.7%, Fig. S1), suggesting N66's basal position. Furthermore, N59 and N60 were rejected as potential ancestral haplotypes by the Shimodaira-Hasegawa (SH) test (∆lnL = 1.054, P < 0.001). Median-joining network (MJN) analysis yielded a star-like network without an obvious geographic structure. Some newly derived terminal haplotypes were generally endemic or private, and interior haplotypes were widely distributed and shared across different regions (Fig. 3, Table S1).

### Genetic diversity and population differentiation

Among the four geographic groups defined (see Methods), decreasing trends in genetic diversity and the proportion of private haplotypes were revealed from the South China Sea group (IV: H/Π, 0.991/3.19%; Np/N, 0.78) through the coastal East China group (Ш: H/Π, 0.978/2.61%; Np/N, 0.54) to the coastal Japan group (І: H/Π, 0.819/2.22%; Np/N, 0.31) (Tables 1, S1). Significant genetic differentiation (Bonferroni correction, P < 0.017) based on the haplotype frequency and the TrN + G distance was detected between the coastal Japan group (I) and China groups (III and IV; Table 2). Furthermore, a high endemism index within these three groups (0.77–0.88) also indicated their historical isolations and limited migration. However, high gene flow (Nm>1) was observed in all pairwise comparisons among three groups (I, III and IV).

### Demographic analyses

A unimodal curve was observed in mismatch distribution analyses for all sequences of *P. modestus* (Fig. 4), and it was not significantly different from the expected sudden expansion model (SSD = 0.003, P = 0.672). The population expansion of *P. modestus* was also supported by two neutrality tests (Tajima’s D = −1.923, P = 0.005 and Fu’s Fs = −24.992, P = 0). Based on expansion parameter τ (τ = 7.438), the expansion time was inferred to be 248 thousand years ago (kya).

Bayesian Skyline Plot (BSP) presented a more detailed demographic history (Fig. 5b). The time to the recent common ancestor (TMRCA) of *P. modestus* was estimated to be 365 (539–210) kya, consistent with the inferred population expansion time (248 kya). A slow population growth during 365–120 kya and a slight bottleneck during 120–70 kya followed by rapid expansion were revealed after 70 kya. The plot of the East China Sea group showed a relative constant population size for a long time and a recent rapid expansion after 60 kya (Fig. 5b). The BSP of the South China Sea group revealed a slow population growth in 350–140 kya and a slight bottleneck in 140–100 kya followed by rapid expansion after 100 kya (Fig. 5b). Through comparison, the South China Sea group showed an older TMRCA and earlier expansion than the East China Sea group. Furthermore, the effective population size in the South China Sea was higher than that in the East China Sea since 300 kya.

## Discussion

**Figure 1:**
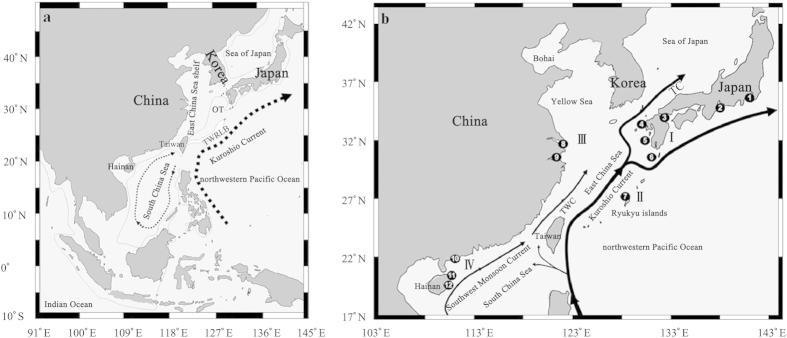
The sampling sites of *P. modestus* and oceanographic conditions in the China seas during the glacial^53^,^54^ (a) and contemporary^39^,^40^ (b) periods. Two glacial sea basins (Okinawa Trough (OT) and South China Sea) were isolated by the exposure of the Taiwan Strait and the Taiwan-Ryukyu land bridge (TWRLB). The glacial shoreline and ocean current are denoted by the light solid line and dotted arrows, respectively. The contemporary surface circulations, including the summer Southwest Monsoon Current, the Taiwan Warm Current (TWC), and the main course of the Kuroshio Current and Tsushima Current (TC), are shown by solid arrows. The names of the numbered sampling localities and four geographic groups (I, II, III and IV) corresponding to different regions are listed in Table 1. The map was created in the Generic Mapping Tools (GMT v5.1.2; http://gmt.soest.hawaii.edu/) software package.

**Figure 2:**
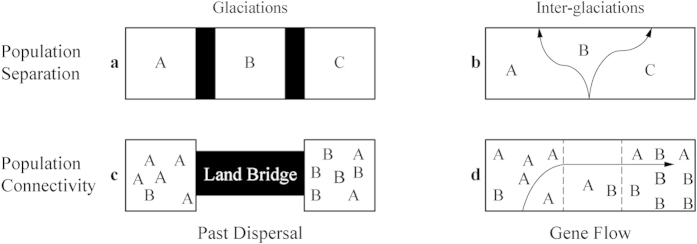
Evolutionary patterns of marine organisms in the China seas: Regional vicariance by glacial sea basins (a) or postglacial sea currents (b) and population connectivity through glacial dispersal (c) or present-time gene flow driven by sea currents (d). Black boxes indicate an exposed shallow shelf, and white boxes indicate regional populations (**A–C**). The arrows mark sea currents.

The genetic signal of glacial isolation was suggested by high endemism indexes within the coastal East China (III, 0.77) and the South China Sea (IV, 0.88) groups (Table 1). These glacial isolated sea basins would have caused private haplotypes, heterogeneous populations or divergent lineages between the East China Sea (Okinawa Trough) and the South China Sea^20^,^23^,^24^ due to the exposure of the Taiwan Strait (Figs. 1a, 2a). However, the physical separation was likely disturbed by a fluctuating sea level. The postglacial rising sea level and the re-flooded Taiwan Strait were likely responsible for the contemporary high gene flow of *P. modestus* (Table 2, Nm>11) between these once fragmented habitats (Fig. 1b). Moreover, a seasonal change in monsoon circulations^38^ could drive planktonic larvae of marine organisms to migrate between the East China Sea and South China Sea^31^ (Fig. 2d).

The significant genetic differentiation and high endemism indexes revealed between/within the geographic groups of coastal Japan (I) and China (III and IV; Tables 1, 2) indicate a negative influence of the contemporary Kuroshio Current on the connectivity of marine organisms in the East China Sea (Fig. 2b). The postglacial Kuroshio Current branches into the East China Sea, the Sea of Japan and the northwestern Pacific regions^39^ (Fig. 1b) and thus divides the sea area into heterogeneous sub-habitats with different temperatures and salinities^40^. The Kuroshio Current was shown to act as a dispersal barrier to promote lineage diversification or population differentiation in some marine organisms^16^,^21^,^22^,^41^,^42^,^43^,^44^,^45^,^46^ (Fig. 2b). In this study, the branches of the Kuroshio Current also seem to have influenced the population structure of *P. modestus*.

**Figure 3:**
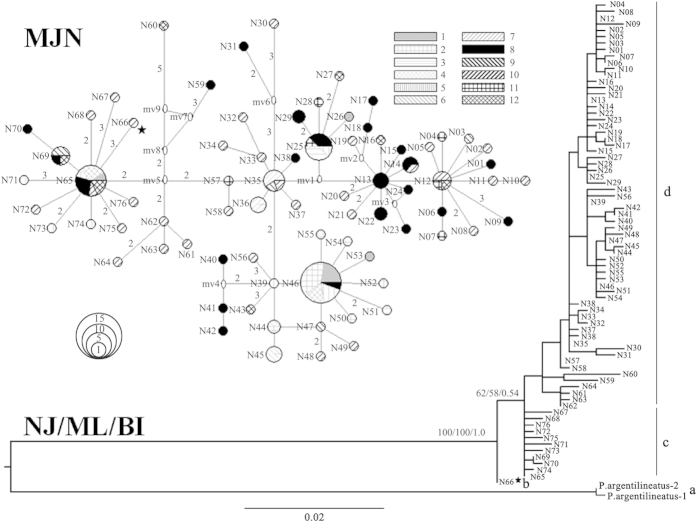
Phylogenetic trees (NJ, ML and BI) and network (MJN) of haplotypes. Sampled haplotypes are indicated by names (N01–N76), and the hollow ovals represent unsampled haplotypes (mv1–9) in MJN. The numbers on the branches show the bootstrap values over 50% (NJ/ML) or posterior probability values over 0.5 (BI) and the number of substitutions over 2 in MJN. The 12 sampling localities are distinguished by different colours (grey for 1–7, black for 8–12) and styles, and the size of the circles is proportional to the frequency of haplotypes in MJN. The basal haplotype N66 is indicated using a star. The four clusters a, b, c, d were shown.

The high gene flow (Nm>1) observed between the coasts of China and Japan (Table 2) likely indicates a past dispersal instead of ongoing migration in the China seas (Fig. 2c). The isolation time (<10 kyr) of *P. modestus* linked to the postglacial Kuroshio Current seems to be insufficient in accumulating a deep genetic divergence between mainland China and the main islands of Japan. Similar historical population dispersals at times of a lower sea level across a long distance were observed in some West Pacific marine taxa^47^,^48^,^49^. Due to the inability to discriminate among contemporary gene flow and historical events in most traditional population structure analyses^50^, the inferred genetic connectivity of *P. modestus* between coastal China and the main islands of Japan might be attributed to a historical coastline connection and a long-distance dispersal instead of contemporary gene flow^51^ (Fig. 2c). The endemic distribution of terminal haplotypes and the sharing of interior haplotypes (Fig. 3, MJN) further indicate historical range expansion^50^ across coastal China, Japan and the Ryukyu Islands. During the Last Glaciation Maximum, the sea level dropped ca. 130–150 m in the East China Sea^52^, and a land bridge connecting Taiwan and the Ryukyu Islands blocked the entry of Kuroshio Current into the East China Sea^53^,^54^ (Fig. 1a). Migration and population admixture became possible when the East China Sea was reduced in size to the elongated Okinawa Trough with a continuous coastline between mainland China and Japan (Figs. 1a, 2c). As an amphibious fish, the larvae of *P. modestus* develop in open water with a planktonic stage of approximately 50 days^55^,^56^. The glacial eastward Kuroshio Current^54^ (Fig. 1a) might also have contributed to its range expansion through larval transport from coastal China to Japan.

**Figure 4:**
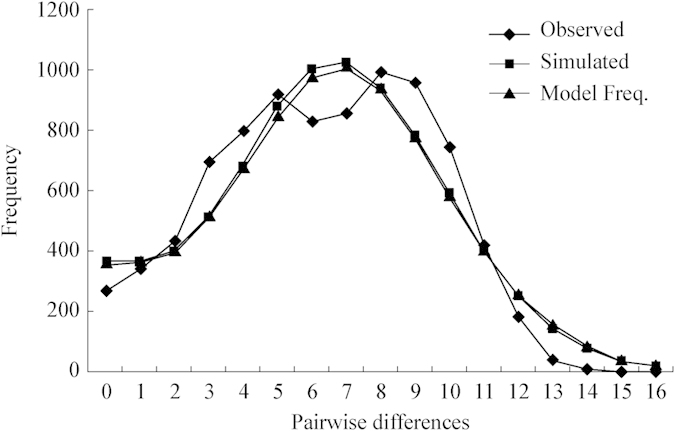
Mismatch distribution analysis for the whole population of *P. modestus*.

**Figure 5:**
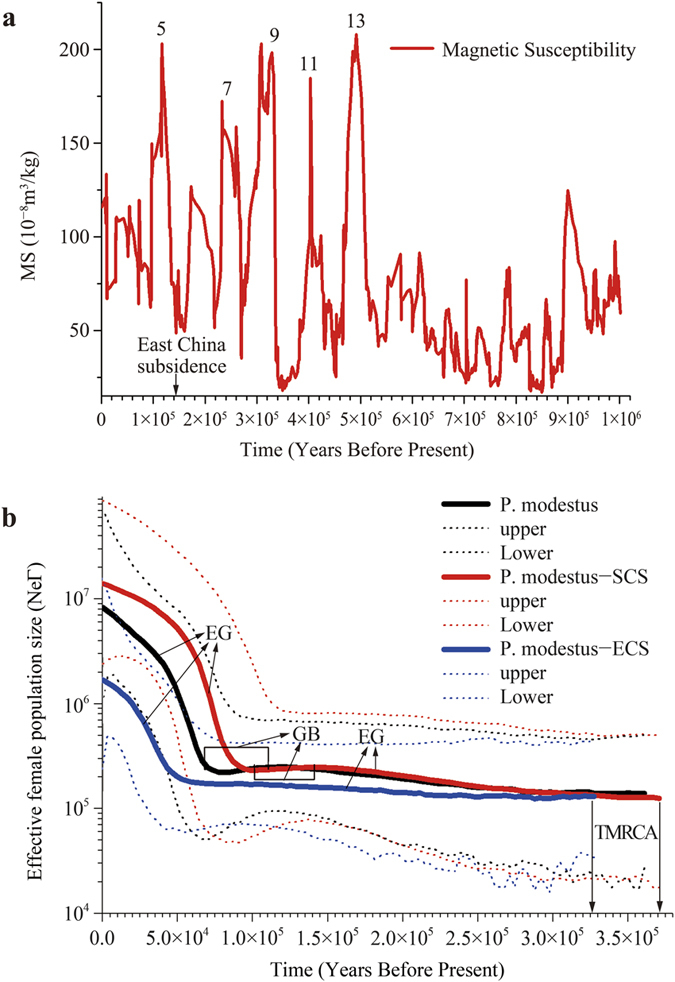
History of the East Asian Summer Monsoon indicated by magnetic susceptibility (MS) 60 **(a) and the demographic history of *P. modestus*, the East China Sea population and the South China Sea population (b).** Thick lines show the medians of population sizes, and thin dotted lines indicate upper and lower 95% credibility intervals. Expansion growth (EG) and genetic bottleneck (GB) are indicated. The last five interglaciations (isotopic stages 5, 7, 9, 11 and 13) are labelled in (**a**).

The signal of the demographic expansion of *P. modestus* was detected through a mismatch distribution analysis (Fig. 4), the two neutrality tests, and BSP (Fig. 5b). The results indicate a rapid population expansion in the whole population of *P. modestus* since ca. 70 kya. Given the uncertainty of the molecular clock, a late Pleistocene expansion since the last interglacial sea-level highstand (<133 kya) can also be inferred for *P. modestus* using a slower molecular rate (e.g., the conventional 1% per million years (/myr) of teleostean mitochondrial *Cyt b* rate^57^). The shoreline enlargement due to East China subsidence occurred in the late Pleistocene^11^,^58^. Furthermore, the strengthening of the East Asian Summer Monsoon in the mid-Pleistocene^59^,^60^ (Fig. 5a) caused high precipitation during the subsequent inter-glaciations and interstades^61^,^62^. The increased rainfall and runoff generally parallelized higher nutrient input into the intertidal habitat^63^, which could have been responsible for late Pleistocene population growth of coastal organisms.

The South China Sea population of *P. modestus* showed a larger historical effective population size and earlier growth relative to the East China Sea population (Fig. 5b). The difference in regional population dynamics is closely related to difference in the primary production between the northern South China Sea and the East China Sea^64^. A decreasing trend in glacial and interglacial mean terrestrial net primary production was observed from coastal South China to East China^65^,^66^,^67^,^68^,^69^,^70^,^71^,^72^. Furthermore, heavier precipitation in low latitude relative to high latitude^73^ can transport much more terrestrial organic matter to intertidal mudflats and coastal regions^74^,^75^. Then, the terrigenous nutrients can be integrated into the food web by benthic microalgae^76^,^77^. Although no obvious global trend in the spatial distribution of intertidal microphytobenthic biomass was revealed at this point, the higher effective population size of mudskipper in the northern South China Sea relative to the East China Sea was likely influenced by regional difference in the intertidal nutrient and primary production^78^. Furthermore, for mudflat-dependent mudskipper, the later population expansion in the East China Sea was likely attributed to the late Pleistocene development of the muddy shoreline derived from the Changjiang Delta^79^,^80^,^81^ due to high microalgae biomass in muddy sediments^82^,^83^. A similar higher population size and earlier expansion were also observed in mitten crabs of *Eriocheir hepuensis* from the coastal northern South China Sea relative to *E. sinensis* from the East China Sea^23^.

Two glacial sea basins (South China Sea and Okinawa Trough) could have served as refugia for marine species in the China seas (Fig. 1a). The question is which one acted as the colonization origin of the species. The present study supports the South China Sea as the origin, based on following clues: First, an older coalescence time (TMRCA) is inferred for the South China Sea population relative to the East China Sea population (Fig. 5b). TMRCA indicates the divergence time within a lineage or population, and the South China Sea population is thus believed to have experienced a longer evolutionary history than the East China Sea population. The difference in the evolutionary time is also consistent with the history of the sea basins: The South China Sea was opened in the Oligocene-middle Miocene (ca. 30–15 million years ago, mya)^84^, whereas the Okinawa Trough formed only in late Miocene and Pliocene (ca. 10–3 mya) as shallow freshwater and brackish water lakes^85^. The reopening and large-scale depression of the Okinawa Trough occurred after the early (<1 mya) and middle Pleistocene (<0.7 mya), respectively^86^,^87^. Although there is uncertainty regarding the evolutionary rate of the mitochondrial ND5 gene, the relative coalescence times of the two geographic populations are apparent. Second, a putative ancestral haplotype (N66) is found only in site 10 from the South China Sea (Figs. 1b, 3 and Table S1). Several other haplotypes (e.g., N65, N67–76) were also inferred as old, close to basal haplotype N66 (Fig. 3). The South China Sea population thus possesses more older haplotypes than the East China Sea population (8 versus 5) even if the sampling size is lower in the former (46 versus 85; Table 1). Third, a descending proportion of private haplotypes from the South China Sea group (IV, 0.78) through the coastal East China group (III, 0.54) to the coastal Japan group (I, 0.31) indicates this species’ colonization origin in the South China Sea because a lower proportion of private haplotypes is expected in the recolonized region^88^. Fourth, genetic diversity is the highest in the South China Sea group (H/Π, 0.991/3.19%) compared to values in the other groups from the East China Sea (e.g., coastal East China group, 0.978/2.61%; coastal Japan group, 0.819/2.22%; and Okinawa group, 0.600/0.09%; Table 1). Similarly, a decreasing trend in genetic diversity from coastal China to Japanese sites is also observed in an estuarine fish (*Salanx ariakensis*)^51^. The colonization origin or glacial refuge is expected to possess higher genetic diversity because of its longer evolutionary time, and the newly colonized regions generally exhibit lower genetic diversity due to the founder effect^5^. Fifth, higher species diversity of the genus *Periophthalmus* occurs in the South China Sea than in the East China Sea (10 versus 3)^36^. Similarly, a higher species diversity of congeners in the South China Sea is observed from the hairtails *Trichiurus*^89^, mud crabs *Scylla*^90^ and fiddler crabs *Uca*^91^. The East Indies Triangle, including the South China Sea, is generally believed to operate as a centre of origin due to the higher species diversity in this area relative to neighbouring regions of the Indo-West Pacific^12^. *P. modestus* is distributed only in the marginal seas of the northwestern Pacific, including the China seas, the Sea of Japan and the eastern Japanese coast^36^. Therefore, through postulating that the South China Sea is the earlier refuge or colonization origin for *P. modestus*, minimum inter-sea basins migration are inferred^92^. *P. modestus* might have dispersed northward into the East China Sea and other marginal seas following the interglacial rising sea level. Subsequently, the northern population would have retreated back into two potential refugia (the Okinawa Trough and the South China Sea) during the period of glaciation. The repeated range expansions and contractions thus caused a gradually declining diversity distribution from the South China Sea through the East China Sea to the coast of Japan.

In summary, this study indicated the negative influence of the postglacial Kuroshio Current and the glacial exposure of the Taiwan Strait on the population structure of *P. modestus*. Although a similar genetic homogeneity was observed among the South China Sea group, the coastal East China Group and the coastal Japan group, contemporary gene flow through the Taiwan Strait and historical dispersal across the Okinawa Trough could be responsible for their population admixture. The demographic history is likely correlated with the mid-Pleistocene strengthened East Asian Summer Monsoon and the difference in primary production between the coastal South China Sea and the East China Sea. As a species distributed in the marginal seas of the northwestern Pacific Ocean, *P. modestus* is inferred to have colonized northward from the South China Sea through the East China Sea to the coasts of Korea and Japan.

## Methods

### Sampling and data collection

A total of 131 individual *P. modestus* fish were evaluated in this study, including those reported by Mukai and Sugimoto^37^. Eighty-three individuals were newly collected from five coastal sites in the East China Sea (sites 8, 9) and the South China Sea (sites 10–12; Fig. 1b) and were preserved in 95% ethanol for molecular analysis. Total genomic DNA was extracted from each specimen using a standard phenol-chloroform extraction method^93^. A segment (approximately 970 bp) of the mitochondrial gene NADH dehydrogenase 5 subunit (ND5) was amplified using the primer pair, L12321-Leu and H13396–ND5M^94^. Initial denaturation was 4 min at 95 °C, followed by 35 cycles of 1 min at 95 °C, 1 min at 55 °C, 2 min at 72 °C, and a final extension of 4 min at 72 °C. PCR products were separated on 1.5% agarose gel and purified with a Gel Extraction Mini Kit (Watson BioTechnologies, Shanghai, China). Purified products were sequenced with the primer H13396 on an ABI Prism 3730 automatic sequencer (Applied Biosystems, Thermo Fisher Scientific Corporation, USA). These sequences were deposited in GenBank with accession numbers HQ453212-HQ453269. ND5 sequences of *P. modestus* and an outgroup species *P. argentilineatus* (GenBank/EMBL/DDBJ accession numbers: AB257605–AB257627) collected from the main islands of Japan and the Ryukyu Islands^37^ were included in the analysis (Table 1). All sequences were aligned using ClustalX^95^ with default parameters. The evolutionary models for the datasets including and excluding outgroups were determined using Modeltest^96^. Two TrN+G models^97^ with different parameters were selected under the Akaike Information Criterion (AIC) for all sequences (gamma = 0.1103; base frequencies A = 0.2845, C = 0.2723, G = 0.1374, T = 0.3057; rate matrix, R[A–C] = R[A–T] = R[C–G] = R[G–T] = 1, R[C–T] = 7.8281, R[A–G] = 12.9280) and hierarchical likelihood ratio tests (hLRTs) for ingroup sequences (gamma = 0.0163; base frequencies A = 0.2831, C = 0.2638, G = 0.1428, T = 0.3104; rate matrix, R[A–C] = R[A–T] = R[C–G] = R[G–T] = 1, R[C–T] = 7.1746, R[A–G] = 13.9593), respectively. A parameters-complicated TIM+I model selection under the AIC for ingroup sequences was not used for subsequent population analyses because of model limitations in those programs.

### Phylogenetic analyses

Four tree-construction methods, NJ^98^, ML^99^, BI^100^ and MJN^101^, were used to recover the intraspecific evolutionary relationship using PAUP (ver. 4.0b10)^102^, MrBayes (ver. 3.2.1)^103^ and NETWORK (ver. 4.613; fluxus-engineering.com), respectively. For NJ analysis, maximum likelihood distances were used. The ML analysis was conducted using a heuristic search with the random addition of sequences (nreps = 10). The nodal supports were assessed using non-parameter bootstrap sampling with 10,000 and 1,000 pseudoreplicates for NJ and ML analysis, respectively. BI was performed with a six-parameter model (GTR+G) similar to TrN+G. These parameters were estimated in the program using the following settings: ngen = 7,000,000; samplefreq = 1000; burnin = 1,750; Nchains = 4; and Nruns = 2. The convergence of independent runs was achieved when white noise was seen in the overlay plot of generation versus the log probability for both runs with the potential scale reduction factor (PSRF) approaching 1 and a low standard deviation of split frequencies (0.005838 < 0.01) after 7,000,000 generations.

Due to uncertainty in the phylogenetic trees, the branching order of the ingroup was assessed based on the inferred phylogenetic tree and network (Fig. 3): (i) The support for the internal branches of four clusters, including a (outgroup), b (N66), c (N65, N67, N68, N69, N70, N71, N72, N73, N74, N75, N76), and d (the rest haplotypes), was estimated using likelihood mapping in TREE-PUZZLE-5.2^104^,^105^. (ii) An alternative scenario to enforce the monophyly of the outgroup and two haplotypes, N59 and N60, with multiple substitutions to others was compared with the estimated ML tree using the SH test^106^ in PAUP and Seq-Gen v1.3.3^107^ with partial optimization and 1000 simulated datasets.

### Population structure analyses

To avoid artificial statistical bias due to a low sample size from some localities, some neighbouring sites were combined into four geographic groups, including the coastal Japan group (I, sites 1–6), the Okinawa Island group (II, site 7), the coastal East China group (III, sites 8 and 9), and the South China Sea group (IV, sites 10–12), in the following population structure analyses, according to some historical (e.g., glacial exposure of the Taiwan Strait) and/or present (e.g., branches of the Kuroshio Current) barriers to gene flow (Fig. 1). The proportion of private haplotypes, the endemism index, the haplotype diversity (H) and the nucleotide diversity (Π) were estimated for each locality and geographic group using ARLEQUIN version 3.5^108^. Excluding insufficient sampling group II, the pairwise genetic divergence (F_ST_ and Φ_ST_) and gene flow (Nm) among three geographic groups (I, Ш and IV) were assessed based on the haplotype frequency and the ingroup’s TrN + G model in ARLEQUIN, respectively. The significance of the F statistics for the geographic group comparisons was evaluated using 10,000 permutations, and the Bonferroni correction for multiple testings^109^ was applied with a lower threshold for the nominal significance level (k = 3, P1 = 0.05/3, and P ≤ 0.017).

### Demographic history

The demographic history of *P. modestus* was inferred through a mismatch distribution analysis^110^ and two neutral tests, Tajima’s D^111^,^112^ and Fu’s Fs^113^, using Arlequin. Both neutrality tests are sensitive to population growth in the absence of selection, and significant negative values generally suggest population expansion^111^,^112^,^113^. The significance of the neutrality tests was assessed in Arlequin by 10,000 permutations. For mismatch analysis, a multimodal distribution is expected for populations in demographic equilibrium, whereas a unimodal distribution usually indicates a recent demographic expansion^110^,^114^. The validity of the estimated stepwise expansion model was tested using the sum of square deviations (SSD) between the observed and expected mismatch as a statistic to infer the significance with the parameter bootstrap approach (10,000 replicates). The expansion time (t) was estimated through the equation t = τ/2 μm, where τ is the mutational timescale, m is the segment length (m = 772 for the present data), and 2 μ is the pairwise mutational rate of the fragment under study. There is no general mitochondrial DNA evolutionary rate for teleosts; an approximate pairwise molecular clock (2 μ) of 3.8%/myr for the ND5 gene from related gobies (*Rhinogobius* species)^32^ was thus used in this study.

A more accurate coalescent model, BSP, implemented in BEAST v1.8.2^115^ and visualized in TRACER v1.6^116^, was also used to estimate the divergence time (TMRCA)^117^ and effective population size changes over time for all sequences of *P. modestus*. Furthermore, the ingroup sequences of *P. modestus* were divided into two geographic groups corresponding to two identified marine eco-regions^118^, the East China Sea (sites 1–9) and the South China Sea (sites 10–12). Subsequently, the population dynamics of two geographic groups from the East China Sea and the South China Sea were further compared using BSP. These analyses were run using the following parameters: 6 × 10^7^ generations, a burn-in of 6 × 10^6^ generations, sampling per 10,000, and 8 groups for the East China Sea; 9 × 10^7^ generations, a burn-in of 9 × 10^6^ generations, sampling per 10,000, and 15 groups for the South China Sea; and 6 × 10^7^ generations, a burn-in of 6 × 10^6^ generations, sampling per 10,000, and 20 groups for all sequences. The effective sample sizes of all runs were over 200. An evolutionary rate (u) of 1.9%/myr^32^ was used to plot population size with respect to time.

## Additional Information

**Accession Codes**: DNA sequence accession numbers: HQ453212-HQ453269 in GenBank.

**How to cite this article**: He, L. *et al*. Biogeographical role of the Kuroshio Current in the amphibious mudskipper *Periophthalmus modestus* indicated by mitochondrial DNA data. *Sci. Rep*. **5**, 15645; doi: 10.1038/srep15645 (2015).

## Supplementary Material

Supplementary Information

## Figures and Tables

**Table 1:** Sampling localities, sample size, number of haplotypes and genetic diversity of *P. modestus*.

Population ID	Locality	Coordinates	N/Nh/Np	Proportion of private haplotypes (Np/N)	Endemism indices (Np/Nh)	H/Π (%)
I (1~6)	Coastal Japan group	−	**42/16/13**	**0.31**	**0.81**	0.819/ 2.22
1	Edogawa, Japan	35˚42′05′′N 139˚55′18′′E	7/4/2	0.29	0.50	0.714/ 1.88
2	Ibigawa, Japan	35˚05′07′′N 136˚41′01′′E	12/6/4	0.33	0.67	0.682/ 0.15
3	Yamagunigawa, Japan	33˚36′29′′N 131˚11′02′′E	6/3/1	0.17	0.33	0.733/ 0.81
4	Imarigawa, Japan	33˚17′16′′N 129˚50′01′′E	4/2/0	0	0	0.500/ 2.79
5	Rokkakugawa, Japan	33˚11′23′′N 130˚12′03′′E	9/7/4	0.44	0.57	0.917/ 3.55
6	Tanegashima Island, Ryukyu Islands	30˚26′29′′N 130˚57′10′′E	4/2/1	0.25	0.50	0.500/ 0.08
II (7)	Okinawajima Island, Ryukyu Islands	26˚39′02′′N 127˚58′33′′E	**6/2/1**	**0.17**	**0.50**	0.600/ 0.09
III (8, 9)	Coastal East China group	−	**37/26/20**	**0.54**	**0.77**	0.978/ 2.61
8	Chongming Island, Shanghai, China	31˚31′30′′N 121˚57′30′′E	32/23/18	0.56	0.78	0.976/ 2.51
9	Nanhui, Shanghai, China	30˚51′36′′N 121˚54′36′′E	5/4/2	0.40	0.50	0.900/ 2.98
IV (10 ~ 12)	South China Sea group	−	**46/41/36**	**0.78**	**0.88**	0.991/ 3.19
10	Hailing Island, Yangjiang, Guangdong, China	21˚39′0′′N 111˚57′36′′E	33/31/27	0.82	0.87	0.996/ 3.46
11	Longlou, Wenchang, Hainan, China	19˚40′48′′N 111˚0′0′′E	6/6/4	0.67	0.67	1.0/ 0.68
12	Dongjiao, Wenchang, Hainan, China	19˚33′36′′N 110˚49′48′′E	7/7/4	0.57	0.57	1.0/ 3.54

N, sampling size; Nh, number of haplotypes; Np, number of private haplotypes; H, haplotype diversity; Π, nucleotide diversity.

See Mukai and Sugimoto^37^ for localities 1–7; localities 8–12 are from this study.

**Table 2:** Pairwise distance (below diagonal) and Nm (above diagonal) between three geographic groups based on haplotype frequency (F_ST_) and Tamura-Nei distance (Φ_ST_). See Table 1 for population ID.

ID	I	III	IV	I	III	IV
		Frequency			TrN + G	
I	0	6.050	5.352	0	3.933	2.858
III	0.076*	0	98.589	0.113*	0	11.074
IV	0.085*	0.005	0	0.149*	0.043	0

^*^Level of significance, P ≤ 0.017 for Bonferroni correction.
