# Supplementary material for: Biogeographical role of the Kuroshio Current in the amphibious mudskipper Periophthalmus modestus indicated by mitochondrial DNA data
Source: Sci Rep. 2015 Oct 28;5:15645. doi: 10.1038/srep15645 (PMC4623607; doi:10.1038/srep15645)

Supplementary Information for

"Biogeographical role of the Kuroshio Current in the amphibious mudskipper *Periophthalmus modestus* indicated by mitochondrial DNA data"

Lijun He[1, 2, *], Takahiko Mukai[3, †], Ka Hou Chu[4, †], Qiang Ma[5], Jing Zhang[1]

[1]State Key Laboratory of Estuarine and Coastal Research, East China Normal University, 200062, Shanghai, China

[2]East China Sea Fisheries Research Institute, Chinese Academy of Fishery Sciences, 200090, Shanghai, China;

[3]Faculty of Regional Studies, Gifu University, 1-1 Yanagido, 501-1193, Gifu, Japan

[4]Simon F. S. Li Marine Science Laboratory, School of Life Sciences, The Chinese University of Hong Kong, Hong Kong, China

[5]Chongming Dongtan National Nature Reserve, 202183, Shanghai, China

[*]Correspondence: Lijun He, State Key Laboratory of Estuarine and Coastal Research, East China Normal University, Shanghai, 200062, China

Tel: 8621-52124974; fax: 8621-62546441; e-mail: tiger02j@hotmail.com

Supplementary Information Table S1:

Geographic distribution and frequency of haplotypes from 12 localities in the northwestern Pacific Ocean

	Coastal Japan (I)						Okinawa (Ⅱ)	Coastal East China (Ⅲ)		South China Sea (Ⅳ)			
Haplotypes	1	2	3	4	5	6	7	8	9	10	11	12	Total
N01								1					1
N02										1			1
N03									1				1
N04											1		1
N05										1			1
N06								1					1
N07											1		1
N08										1			1
N09								1					1
N10										1			1
N11										1			1
N12										2	1	1	**4**
N13								3					**3**
N14								2		1			**3**
N15								1					1
N16												1	1
N17								1					1
N18								1					1
N19										1			1
N20										1			1
N21										1			1
N22								2					**2**
N23								1					1
N24								1					1
N25			3		1			3			1		8
N26	1												1
N27												1	1
N28											1		1
N29								2					**2**
N30										1			1
N31								1					1
N32										1			1
N33										1			1
N34										1			1
N35							3		1	1			5
N36							3						**3**

N37									1			1
N38							1					1
N39					1							1
N40							1					1
N41							1					1
N42							1					1
N43											1	1
N44		1				1						**2**
N45						3						**3**
N46	4	7	2	1	3		1					**18**
N47								1				1
N48									1			1
N49									1			1
N50		1										1
N51			1									1
N52		1										1
N53	1											1
N54		1										1
N55		1										1
N56									1			1
N57										1		1
N58									1			1
N59							1					1
N60											1	1
N61									1			1
N62									1			1
N63									1			1
N64									1			1
N65	1			3	1		3		2		1	11
N66									1			1
N67									1			1
N68									1			1
N69							1	2			1	**4**
N70							1					1
N71					1							1
N72									1			1
N73					1							1
N74					1							1
N75									1			1
N76									1			1

Supplementary Information Figure S1:

Quartets of sequences from four clusters including a (outgroup), b (N66), c (N65, N67, N68, N69, N70, N71, N72, N73, N74, N75, N76), and d (the rest haplotypes) used in the likelihood mapping statistics.

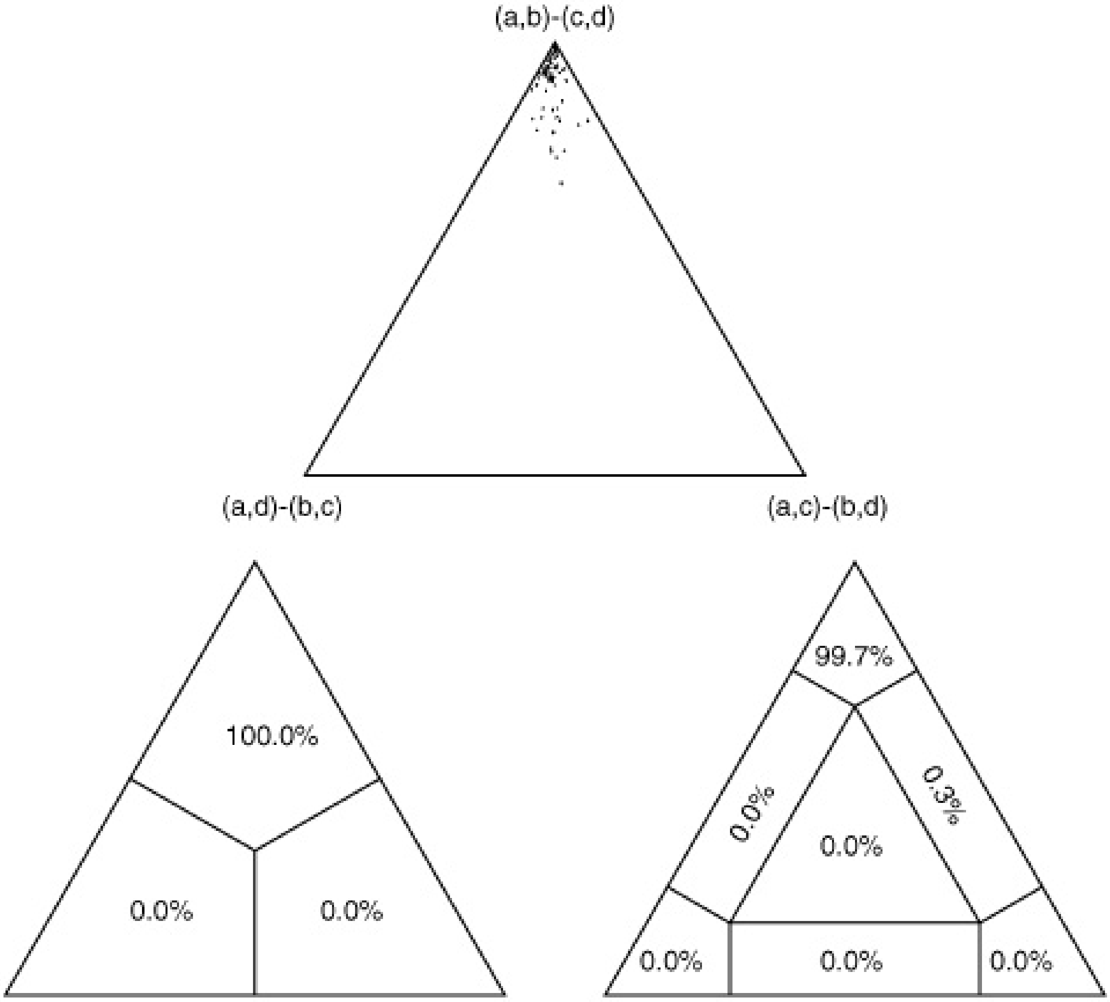

Supplement: Supplementary Information [file srep15645-s1.pdf]
